# ChatGPT-4 Omni Performance in USMLE Disciplines and Clinical Skills: Comparative Analysis

**DOI:** 10.2196/63430

**Published:** 2024-11-06

**Authors:** Brenton T Bicknell, Danner Butler, Sydney Whalen, James Ricks, Cory J Dixon, Abigail B Clark, Olivia Spaedy, Adam Skelton, Neel Edupuganti, Lance Dzubinski, Hudson Tate, Garrett Dyess, Brenessa Lindeman, Lisa Soleymani Lehmann

**Affiliations:** 1 UAB Heersink School of Medicine, 1670 University Blvd, Birmingham, AL, 35233, United States, 1 2566539498; 2 University of South Alabama Whiddon College of Medicine, Mobile, AL, United States; 3 University of Illinois College of Medicine, Chicago, IL, United States; 4 Harvard Medical School, Boston, MA, United States; 5 Alabama College of Osteopathic Medicine, Dothan, AL, United States; 6 UT Southwestern Medical Center, Dallas, TX, United States; 7 Saint Louis University School of Medicine, St. Louis, MO, United States; 8 Medical College of Georgia, Augusta University, Augusta, GA, United States; 9 University of Colorado Anschutz Medical Campus School of Medicine, Aurora, CO, United States; 10 Mass General Brigham, Boston, MA, United States

**Keywords:** large language model, ChatGPT, medical education, USMLE, AI in medical education, medical student resources, educational technology, artificial intelligence in medicine, clinical skills, LLM, medical licensing examination, medical students, United States Medical Licensing Examination, ChatGPT 4 Omni, ChatGPT 4, ChatGPT 3.5

## Abstract

**Background:**

Recent studies, including those by the National Board of Medical Examiners, have highlighted the remarkable capabilities of recent large language models (LLMs) such as ChatGPT in passing the United States Medical Licensing Examination (USMLE). However, there is a gap in detailed analysis of LLM performance in specific medical content areas, thus limiting an assessment of their potential utility in medical education.

**Objective:**

This study aimed to assess and compare the accuracy of successive ChatGPT versions (GPT-3.5, GPT-4, and GPT-4 Omni) in USMLE disciplines, clinical clerkships, and the clinical skills of diagnostics and management.

**Methods:**

This study used 750 clinical vignette-based multiple-choice questions to characterize the performance of successive ChatGPT versions (ChatGPT 3.5 [GPT-3.5], ChatGPT 4 [GPT-4], and ChatGPT 4 Omni [GPT-4o]) across USMLE disciplines, clinical clerkships, and in clinical skills (diagnostics and management). Accuracy was assessed using a standardized protocol, with statistical analyses conducted to compare the models’ performances.

**Results:**

GPT-4o achieved the highest accuracy across 750 multiple-choice questions at 90.4%, outperforming GPT-4 and GPT-3.5, which scored 81.1% and 60.0%, respectively. GPT-4o’s highest performances were in social sciences (95.5%), behavioral and neuroscience (94.2%), and pharmacology (93.2%). In clinical skills, GPT-4o’s diagnostic accuracy was 92.7% and management accuracy was 88.8%, significantly higher than its predecessors. Notably, both GPT-4o and GPT-4 significantly outperformed the medical student average accuracy of 59.3% (95% CI 58.3‐60.3).

**Conclusions:**

GPT-4o’s performance in USMLE disciplines, clinical clerkships, and clinical skills indicates substantial improvements over its predecessors, suggesting significant potential for the use of this technology as an educational aid for medical students. These findings underscore the need for careful consideration when integrating LLMs into medical education, emphasizing the importance of structured curricula to guide their appropriate use and the need for ongoing critical analyses to ensure their reliability and effectiveness.

## Introduction

### Overview

Recent studies have demonstrated the promise of large language models (LLMs) such as ChatGPT, Google Gemini, and Claude in various medical applications, with studies showing passing United States Medical Licensing Examination (USMLE) exam scores and evaluating LLMs’ ability to assist with clinical documentation and provide medical advice [1-4]. The potential of these models to revolutionize medicine and medical education underscores the need for a thorough evaluation of their performance [5,6]. Before LLMs can be widely adopted in health care and medical education, it is crucial to assess their proficiency in both preclinical disciplines (eg, anatomy, physiology, and microbiology) and clinical disciplines (eg, diagnostics and treatment recommendations).

### The Role of LLMs in Medical Education

In the context of undergraduate medical education, LLMs have demonstrated preliminary potential in text-based applications in generating practice questions, fostering case-based learning, creating study guides, and providing rapid answers to relevant questions [7-9]. Although models such as GPT-3.5 offer the potential for a more personalized learning experience, they also have limitations, such as training cut-off dates, limited image capabilities, potential inaccuracies, and a lack of user training [10-12]. Medical students often use third-party resources to supplement their studies, with evidence suggesting that such utilization is associated with higher USMLE scores [13,14]. The diverse applications and benefits of LLMs contribute to a comprehensive approach to fostering self-directed learning for lifelong learners in medicine [12,15]. While accuracy remains a limitation of LLMs as clinical tools for students and clinicians, recent studies indicate a trend toward increased reliability and accuracy, a crucial consideration for their use in medical education and health care [16-18].

### Previous Assessments of LLM Accuracy in Medical Contexts

Comparing multiple studies on the accuracy of LLMs in the context of medicine, such as ChatGPT, is challenging due to variations in question sets, exclusion criteria, and the specific models assessed, though some parallels can be drawn. Most studies have evaluated LLMs based on their ability to correctly answer multiple-choice questions (MCQs) from retired National Board of Medical Examiners’ (NBME) content or third-party question banks such as Amboss [19-22]. Some studies suggest LLMs perform better on USMLE sample items compared to third-party question banks [20], and newer versions of LLMs such as ChatGPT 4 (GPT-4) outperform their earlier counterparts [22]. Evaluations of ChatGPT 3.0 found it was able to accurately answer USMLE sample items 36.7% of the time [23], improving to more than 50% correct in a matter of months [21]. Performance also appears to depend on the specific skills tested and the language used in training [24,25]. Further illustrating this in a study by the NBME, ChatGPT scored a passing score in USMLE Step exams across multiple attempts, with one exception in a USMLE Step 3 exam attempt [26]. ChatGPT 3.5 (GPT-3.5) was found to answer 63.06% of Step 1 and 70.0% of Step 2 CK questions correctly [26]. Most recent studies showcase GPT-4 achieving as high as 86% accuracy on USMLE Step 1 questions, highlighting its near readiness for investigation in improving learning for medical students in preclinical education.

### Aim of the Study

While previous research has primarily explored the ability of these models to pass medical licensing exams, this study takes a medical disciplinary approach to assess and compare the accuracy of ChatGPT 3.5 (GPT-3.5), ChatGPT 4 (GPT-4), and ChatGPT 4 Omni (GPT-4o) specifically in the context of the USMLE preclinical medical disciplines and clinical clerkships. These historically recognized USMLE (and NBME [27]) preclinical medical disciplines, including anatomy, pathology, and biochemistry, provide a valuable empirical framework to understand the strengths and weaknesses of language models in medical disciplines and clinical skills.

## Methods

### LLMs: The ChatGPT Series

In our study, we used the ChatGPT series, which comprises sophisticated algorithms designed to simulate human-like responses to textual inputs. These models generate responses by analyzing input text and predicting output based on learned statistical patterns. ChatGPT 3.5 (GPT-3.5) is the earliest model used in this study and is currently accessible to the public through free subscription [28]. ChatGPT 4 (GPT-4), introduced in March 22, 2023 and available through a monthly paid subscription, was included for comparative analysis [29]. Notably, we included the latest ChatGPT model, ChatGPT 4 Omni (GPT-4o), which was released on May 13, 2024 [30].

### Clinical Vignette-Based Assessment in USMLE Disciplines and Clinical Clerkship

In total, 750 clinical vignette-style MCQs were sourced from various question banks provided by medical schools to medical students (Amboss, UWorld, TrueLearn). To prevent model “learning” effects and avoid potential bias from prior usage of publicly available question sets, we selected these MCQs from these sources, which are not publicly accessible.

The 750 MCQs were divided evenly, with 375 covering USMLE Step 1 (“Preclinical”) content and 375 covering USMLE Step 2 (“Clinical”). We applied specific criteria to ensure the relevance and rigor of the questions. Questions involving imaging findings (such as X-rays, MRIs, or ultrasounds), histologic, and gross exam findings were excluded from the study, and an additional clinical vignette was generated in its place. To ensure diversity and reduce bias, questions were sourced by generating random question sessions, with careful attention to avoid duplication of any questions in the final set.

For each MCQ, we noted whether the vignette pertained to preclinical or clinical subject matter, identified the specific USMLE preclinical discipline or clinical clerkship content assessed, and the percentage of medical students who answered correctly, as detailed by the question bank resources. Using the percentage of medical students who correctly answered each question, we assigned a difficulty tier to each question on a scale from 1 (most difficult) to 5 (easiest) (1: 0%‐19.9%; 2: 20.0%‐39.9%; 3: 40.0%‐59.9%; 4: 60.0%‐79.9%; 5: 80.0%‐100%).

### Protocol for Assessing Accuracy of ChatGPT

The assessment of the language models was conducted from May 20 to May 26, 2024. The assessment of response accuracy entailed entering the MCQs into a ChatGPT chat session using a standardized protocol based on methodologies similar to those employed in multiple-choice-based language model assessments [16,17,19,26,31-35]. This protocol for eliciting a response from ChatGPT was as follows: “Answer the following question and provide an explanation for your answer choice.” Data procured from ChatGPT included its selected response, the rationale for its choice, and whether the response was correct (“accurate” or “inaccurate”). Responses were deemed correct if ChatGPT chose the correct multiple-choice answer. To prevent memory retention bias, each vignette was processed in a new chat session.

### Assessment in Clinical Domains of Diagnostics and Management

Further subcategorization of the 750 MCQs was made based on their question stem. Question stems assessing the most likely diagnosis (n=168, “Diagnostics”) or the next best step in treatment (n=178, “Management”) were noted and used for further comparison to assess accuracy in the clinical skills of diagnostics and management.

### Statistical Analysis

IBM SPSS Statistics 29.0 (IBM Corporation) was used for statistical analyses, with a significance threshold of *P*<.05. Statistical tests included chi-squared for categorical comparisons, and binary logistic regression when assessing the influence of question difficulty on language model correct response rate.

### Ethical Considerations

The study did not involve patient data or human subjects and, as such, was not subject to institutional review board approval.

## Results

**Figure 1.**
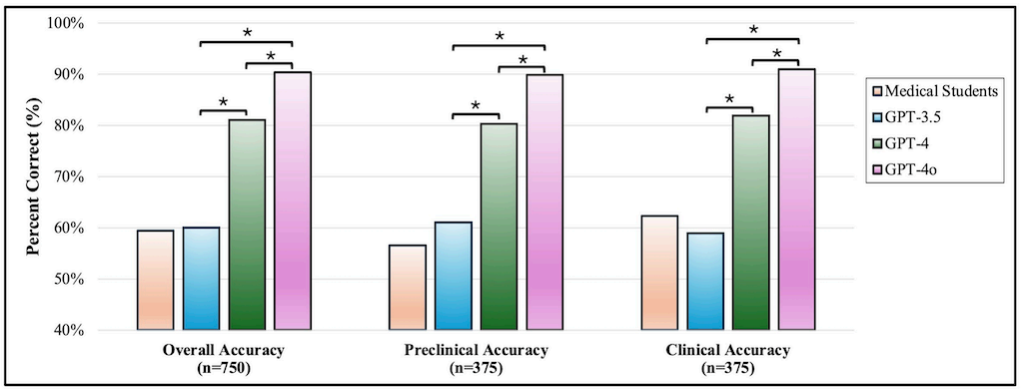
Analysis of ChatGPT models’ and medical students’ performance on USMLE questions. This figure displays the comparative accuracies of ChatGPT 3.5 (GPT-3.5), ChatGPT 4 (GPT-4), ChatGPT 4 Omni (GPT-4o), and medical students in answering a set of 750 USMLE-style questions. The overall accuracy, preclinical accuracy, and clinical accuracy are shown. Asterisks (*) denote statistically significant differences (*P*<.05), highlighting the advancements in newer models of the GPT series. The number of questions is indicated for each category: n=750 for overall accuracy, n=375 for preclinical accuracy, and n=375 for clinical accuracy. GPT: Generative Pre-trained Transformer; USMLE: United States Medical Licensing Examination.

Overall, GPT-4o achieved an overall correct response rate of 90.4%, while GPT-4 had 81.1%, both significantly outperforming GPT-3.5’s correct response rate of 60.0% (Table 1 and Figure 1). The average accuracy of medical students was 59.3% (95% CI 58.3‐60.3).

**Table 1.** Response accuracy of the ChatGPT series across USMLE^a^ preclinical disciplines and clinical clerkships. Some questions (n=139) could not be categorized due to not having or having multiple categories from sources.

Question category or subcategory	Questions, N	Language model performance, n (%) correct			Medical student average, percent correct (95% CI)
		GPT^b^-3.5	GPT-4	GPT-4o	
Overall					
All questions	750	450 (60.6)	608 (81.1)	678 (90.4)	59.3 (58.3‐60.3)
Preclinical assessment questions					
All questions	375	229 (61.1)	301 (80.3)	337 (89.9)	57.7 (56.3‐59.1)
USMLE disciplines					
Anatomy, histology, and embryology	36	21 (58.3)	31 (86.1)	31 (86.1)	50.7 (45.9‐55.5)
Behavioral and neuroscience	52	40 (76.9)	45 (86.5)	49 (94.2)	53.3 (47.8‐58.8)
Biochemistry	35	20 (57.1)	28 (80.0)	31 (88.6)	65.1 (57.8‐72.3)
Biostatistics	21	12 (57.1)	18 (85.7)	17 (81.0)	57.1 (52.7‐61.6)
Immunology	28	19 (67.9)	23 (82.1)	26 (92.9)	53.5 (48.1‐58.9)
Microbiology	39	20 (51.3)	30 (76.9)	36 (92.3)	57.7 (52.0‐63.2)
Pathology	29	17 (58.6)	20 (69.0)	24 (82.8)	64.4 (60.9‐67.8)
Pharmacology	44	27 (61.3)	37 (84.1)	41 (93.2)	57.9 (53.8‐62.0)
Physiology	24	13 (54.2)	12 (50.0)	20 (83.3)	51.9 (46.1‐57.8)
Social sciences	22	13 (59.1)	18 (81.8)	21 (95.5)	66.7 (61.5‐72.1)
Clinical assessment questions					
All questions	375	221 (58.9)	307 (81.9)	341 (90.9)	61.0 (59.5‐62.5)
Clinical clerkships					
Family medicine	34	20 (59.0)	26 (76.5)	34 (100.0)	54.0 (48.4‐59.5)
Internal medicine	22	15 (68.2)	21 (95.5)	22 (100.0)	69.2 (65.1‐73.2)
Neurology	59	41 (69.5)	50 (84.7)	55 (93.2)	61.2 (57.2‐65.3)
Obstetrics and gynecology	45	24 (53.3)	40 (88.9)	41 (91.1)	61.2 (54.9‐67.6)
Pediatrics	42	28 (66.7)	32 (76.2)	37 (88.1)	58.3 (54.2‐62.5)
Psychiatry	43	25 (58.1)	35 (81.4)	40 (93.0)	54.2 (48.5‐59.8)
Surgery	36	20 (55.6)	30 (83.3)	31 (86.1)	62.3 (57.4‐67.1)

a USMLE: United States Medical Licensing Examination.

b GPT: Generative Pre-trained Transformer.

### USMLE Discipline Response Accuracies

In total, 375 MCQs designed to assess preclinical content as categorized by USMLE disciplines were administered to GPT-3.5, GPT-4, and GPT-4o. GPT-3.5’s highest correct response percentages were in behavioral and neuroscience (76.9%), immunology (67.9%), and pharmacology (61.3%). Conversely, the lowest correct response percentages were observed in physiology (54.2%) and microbiology (51.3%). For GPT-4, the highest correct response percentages were observed in behavioral and neuroscience (86.5%), anatomy, histology, and embryology (86.1%), and pharmacology (84.1%). The lowest correct response percentages for GPT-4 were in physiology (50.0%) and pathology (69.0%). GPT-4o demonstrated the highest correct response percentages in social sciences (95.5%), behavioral and neuroscience (94.2%), and pharmacology (93.2%). The lowest correct response percentages for GPT-4o were in pathology (82.8%) and biostatistics and epidemiology (81.0%).

### Response Accuracies in Clinical Clerkships

In total, 375 MCQs assessing clinical clerkship content were administered to GPT-3.5, GPT-4, and GPT-4o. GPT-3.5 exhibited its highest response percentages in neurology (69.5%) and internal medicine (68.2%), while the lowest percentage response accuracies were observed in obstetrics and gynecology (53.3%) and surgery (55.6%). In comparison, GPT-4 achieved higher accuracy across all clerkships, with notable performances in internal medicine (95.5%) and obstetrics and gynecology (88.9%). Similarly, GPT-4o demonstrated improved performance, achieving correct response rates of 93.2% in neurology and 93.0% in psychiatry, as well as 100.0% in family medicine and 100.0% in internal medicine. The lowest accuracies for GPT-4o were still significantly high, with obstetrics and gynecology at 91.1% and surgery at 86.1%. Overall, GPT-4 and GPT-4o showed substantial improvements over GPT-3.5 in all clinical clerkship categories.

### Vignette Difficulty and Comparisons Based on Respondent Performance

**Figure 2.**
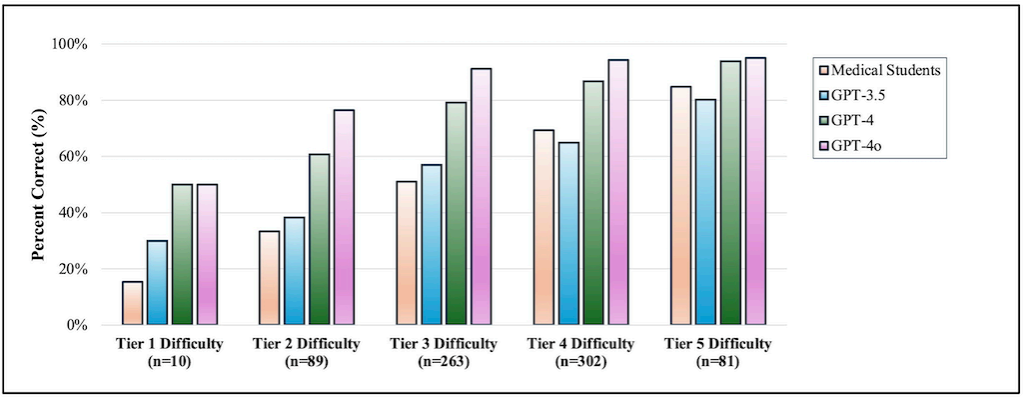
Influence of question difficulty on response accuracy compared to medical student performance. This figure illustrates the effect of clinical vignette difficulty on the response accuracy of ChatGPT 3.5 (GPT-3.5), ChatGPT 4 (GPT-4), and ChatGPT 4 Omni (GPT-4o) in comparison to medical students. The bar graph represents the percentage of correct responses across different tiers of difficulty, ranging from tier 1 (most difficult) to tier 5 (easiest). The number of questions for each difficulty tier is n=10 for tier 1, n=89 for tier 2, n=263 for tier 3, n=302 for tier 4, and n=81 for tier 5.

GPT-3.5 (Exp(B)=1.033, SE=0.005, *P*<.001), GPT-4 (Exp(B)=1.039, SE=0.006, *P*<.001), and GPT-4o (Exp(B)=1.043, SE=0.008, *P*<.001) demonstrated a higher likelihood of responding incorrectly to vignettes that were more challenging for medical student respondents (Figure 2).

### Performance of ChatGPT in Diagnostics and Management

**Figure 3.**
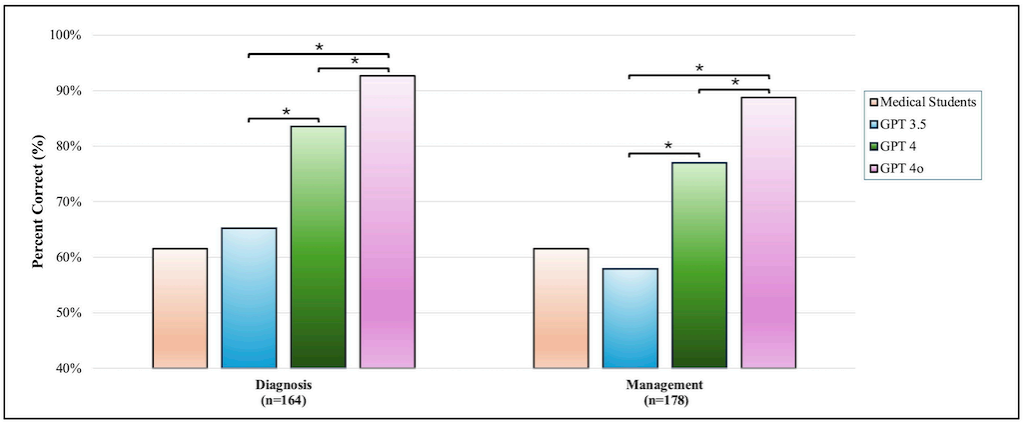
Performance of ChatGPT models in diagnostics and management compared to medical students. This figure compares the performance of ChatGPT 3.5 (GPT-3.5), ChatGPT 4 (GPT-4), and ChatGPT 4 Omni (GPT-4o) in the clinical domains of diagnostics and management. The bar graph shows the percentage of correct responses for each model and medical students in the diagnosis (n=164) and management (n=178) categories. GPT-4o exhibits the highest accuracy in both categories, followed by GPT-4, with GPT-3.5 showing the lowest performance. Asterisks (*) denote statistically significant differences (*P*<.05), emphasizing the advancements in newer models of the GPT series. GPT: Generative Pre-trained Transformer.

A total of 342 MCQs were secondarily categorized from the 750 MCQs based on question stems: 164 assessing “diagnostics” and 178 assessing “management.” Overall, the respective percent correct response accuracies of GPT-3.5, GPT-4, and GPT-4o in these questions were 70.5% (241/342), 81.9% (280/342), and 88.8% (304/342) (Figure 3). In the diagnostics category, GPT-4 and GPT-4o demonstrated higher correct response percentages compared to GPT-3.5 (83.5% and 92.7% vs 65.2%). Similarly, in the management category, GPT-4 and GPT-4o outperformed GPT-3.5 (77.0% and 88.8% vs 57.9%). Notably, GPT-4o significantly outperformed GPT-4 in both diagnostics and management.

## Discussion

### Overview

This study evaluated ChatGPT versions for their accuracy in USMLE preclinical disciplines, clinical clerkships, and clinical skills categories of diagnostics and management. The aim was to assess the reliability of using LLMs in medical education by examining their accuracy across various preclinical and clinical disciplines. Dependable accuracy in these areas underlies the potential of LLMs to support medical education effectively. Our findings highlighted varied performances across disciplines, with a significant increase in response accuracy observed for GPT-4o over GPT-4 and GPT-3.5.

### Overall Performance and Disciplinary Accuracies

Overall, GPT-4o achieved an accuracy rate of 90.4%, significantly outperforming both GPT-3.5 (60.0%) and GPT-4 (81.1%). This improvement is consistent across both preclinical and clinical domains, emphasizing the advancements in model development. GPT-4o’s highest preclinical accuracy rates were observed in social sciences (95.5%), behavioral and neuroscience (94.2%), and pharmacology (93.2%). In clinical clerkships, GPT-4o maintained high accuracy, particularly in family medicine and internal medicine, where it achieved a 100% correct response rate, and demonstrated strong performance in neurology and psychiatry. These findings underline GPT-4o’s potential utility in medical education and emphasize the necessity of its strategic integration into educational curricula.

### Question Difficulty and Comparison With Medical Student Performance

Notably, there was a significant positive correlation between the percentage of correct responses by medical students and the likelihood of correct responses by the LLMS, which indicates that as vignette difficulty increased, the performance of the artificial intelligence (AI) models reflected the difficulty gradient experienced by the students. However, it is worth noting that GPT-4o achieved an overall accuracy of 90.4% in a question set where the medical students average was less than that of a passing USMLE exam score (59.3%).

### Improvements in Diagnostics and Management

The clinical vignette-based assessments further illustrated the improvements in GPT-4o in diagnostics and management. In diagnostics, GPT-4o achieved a 92.7% accuracy rate, surpassing GPT-4 (83.5%) and GPT-3.5 (65.2%). Similarly, in management tasks, GPT-4o’s accuracy was 88.8%, significantly higher than both GPT-4 (77.0%) and GPT-3.5 (57.9%).

### Factors Contributing to Improved Performance

The improvements seen in GPT-4o could be attributed to several advancements in its architecture and the model’s training, such as more comprehensive datasets and refined algorithms. This trend of improvement aligns with previous research noting the progressive enhancements in LLMs’ accuracy and reliability [16-18]. However, an important consideration is the potential interaction between LLM performance and the Flynn effect, which describes the observed rise in intelligence test scores over time. As LLMs are trained on increasingly up-to-date data, they may reflect or even amplify these trends, potentially impacting the psychometric validity of assessments like the USMLE. For instance, environmental influences and the availability of more recent data can significantly impact cognitive performance, a factor that may similarly affect AI models [36]. The implications of this interaction warrant further exploration, as understanding these dynamics could provide valuable insights into both the short-term and long-term reliability of LLM-assisted test performance in medical education. Additionally, the recency of the datasets used to train GPT-4 and GPT-4o could be another factor contributing to their improved accuracy compared to GPT-3.5. As these improvements continue, it is essential to assess how they contribute not only to immediate gains in performance but also to the broader implications for long-term educational outcomes and assessment integrity.

### Considerations for Integration in Medical Education

Several considerations must be addressed before integrating these models into medical education. The ability to correctly answer USMLE questions is not necessarily the same as synthesizing and reasoning about a patient’s history, clinical symptoms, physical exam findings, and laboratory data. This raises the concern of whether LLMs will be able to provide safe and accurate guidance to clinicians at the bedside who are struggling to make sense of a patient’s illness. It will therefore be important to assess the value of LLMs in real clinical situations and to assess if and how they can be safely deployed in clinical settings. To address this, medical schools and residency program directors should establish mechanisms to continuously monitor the performance and impact of LLMs used in clinical settings. It would be valuable to create a national registry of feedback from students and faculty to identify errors and unintended consequences associated with the use of LLMs in medical education and clinical care.

In the context of American medical education, standardized testing environments such as the USMLE play a critical role in shaping the applicability of LLMs like GPT-4o. These models must adapt to a testing culture that heavily emphasizes MCQ formats, which are integral to medical training and licensure in the United States. While LLMs offer potential advantages, there is a risk that over-reliance on AI could hinder the development of essential diagnostic skills in medical students and clinicians [37,38]. This dependency on AI tools may lead to a decline in critical thinking and problem-solving abilities, particularly in situations where AI support is unavailable [39,40]. These concerns underscore the importance of thoughtfully integrating AI into medical education, with careful consideration of its long-term impact on clinical competencies and ethical implications, such as fairness and equity in training future health care professionals [37,38].

### Ethical Implications of AI Integration With Medical Education

The ethical implications of integrating AI, including LLMs, in medical education and patient care require thorough consideration. Issues such as data privacy, the potential for systemic bias in AI algorithms, and the lack of accountability in AI-driven decisions pose serious challenges. The inherent biases in training data can lead to skewed AI responses, impacting clinical decision-making processes [41]. Moreover, the reliance on AI-driven tools raises concerns about the equitable distribution of these technologies, as access often requires paid subscriptions, which could exacerbate disparities in medical education. To mitigate these risks, educational institutions should implement clear guidelines for AI use, including regular audits of AI performance and mandatory training for students and faculty on the limitations and ethical considerations of AI tools. Additionally, establishing dedicated oversight committees to monitor AI integration and address any emerging issues in real-time will be crucial to ensuring these technologies are used responsibly and effectively.

### Study Limitations

This study contains several limitations. The 750 MCQs are robust, although they are “USMLE-style” questions and not actual USMLE exam questions. The exclusion of clinical vignettes involving imaging findings limits the findings to text-based accuracy, which potentially skews the assessment of disciplinary accuracies, particularly in disciplines such as anatomy, microbiology, and histopathology. Additionally, the study does not fully explore the quality of the explanations generated by the AI or its ability to handle complex, higher-order information, which are crucial components of medical education and clinical practice—factors that are essential in evaluating the full utility of LLMs in medical education. Previous research has highlighted concerns about the reliability of AI-generated explanations and the risks associated with their use in complex clinical scenarios [10,12]. These limitations are important to consider as they directly impact how well these tools can support clinical reasoning and decision-making processes in real-world scenarios. Moreover, the potential influence of knowledge lagging effects due to the different datasets used by GPT-3.5, GPT-4, and GPT-4o was not explicitly analyzed. Future studies might compare MCQ performance across various years to better understand how the recency of training data affects model accuracy and reliability.

### Future Research Directions

Future research should aim to expand the analysis of medical education to incorporate more diverse clinical vignettes, especially those involving imaging and other multimedia content. This would provide a more comprehensive assessment of LLM capabilities. Longitudinal studies are also needed to evaluate the long-term effects of AI integration on learning outcomes and clinical decision-making skills. Moreover, investigating methods to mitigate inherent biases in LLMs and exploring the integration of AI with traditional educational methodologies could provide a more balanced view of the potential and limitations of these technologies in medical training.

### Conclusions

In conclusion, this study provides an assessment of the response accuracies of the ChatGPT series across a wide array of USMLE preclinical disciplines and clinical clerkships. The significant improvements observed in ChatGPT 4 Omni suggest substantial potential for its use as a tool for medical education. As the utilization of AI by medical students and clinicians increases, our findings emphasize the need for formal curricula and guidelines that ensure proper usage, as well as the necessity of robust validation and oversight processes for LLMs as they are integrated into medical education.

## Abbreviations

AI: artificial intelligence
GPT-3.5: ChatGPT 3.5
GPT-4: ChatGPT 4
GPT-4o: ChatGPT 4 Omni
LLM: large language model
MCQ: multiple-choice question
NBME: National Board of Medical Examiners
USMLE: United States Medical Licensing Examination
